# Determinants of quality contraceptive counselling information among young women in Sierra Leone: insights from the 2019 Sierra Leone demographic health survey

**DOI:** 10.1186/s12905-023-02419-8

**Published:** 2023-05-15

**Authors:** Quraish Sserwanja, Lilian Nuwabaine, Kassim Kamara, Milton W. Musaba

**Affiliations:** 1 Programmes Department, GOAL Global, Arkaweet Block 65 House No. 227, Khartoum, Sudan; 2 School of Nursing and Midwifery, Aga Khan University, Kampala, Uganda; 3 National Disease Surveillance Programe, Ministry of Health and Sanitation, Free town, Sierra Leone; 4 Department of Obstetrics and Gynaecology, Busitema University/ Mbale Regional Referral and Teaching Hospital, Mbale, Uganda; 5 Busitema University Centre of Excellence for Maternal Reproductive and Child Health (BuCEMaRCH), Mbale, Uganda

**Keywords:** Young people, Family planning, Quality, Counselling, Sierra Leone

## Abstract

**Background:**

The quality of contraceptive counseling information received by prospective clients of family planning services can greatly influence both the uptake and continued use of contraceptives. Therefore, an understanding of the level and determinants of quality contraception information among young women in Sierra Leon could inform family programs, with the aim of reducing the high unmet need in the country.

**Methods:**

We analyzed secondary data from the 2019 Sierra Leone Demographic Health Survey (SLDHS). Participants were young women aged 15–24 years, who were using a family planning method (*n* = 1,506). Good quality family planning counselling was defined a composite variable that included; a woman being told about side effects, how to deal with side effects, and the availability of other family planning methods/options. Logistic regression was performed using SPSS software version 25.

**Results:**

Out of 1,506 young women, 955 (63.4%, 95% CI: 60.5–65.3) received good quality family planning counselling services. Of the 36.6% that received inadequate counselling, 17.1% received no counselling at all. Good quality family planning counselling services was positively associated with receiving family planning services from government health facilities (aOR: 2.50, 95% CI: 1.83–3.41), having no major challenges with distance to access healthcare (aOR: 1.45, 95% CI: 1.10–1.90), having visited a health facility (AOR: 1.93, 95% CI: 1.45–2.58), and having been visited by a health field worker within the last 12 months (aOR: 1.67, 95% CI: 1.24–2.26) while residing in the southern region ( aOR: 0.39, 95% CI: 0.22–0.69) and belonging to the richest wealth quintile (aOR: 0.49, 95% CI: 0.24–0.98) were associated with less odds of receiving good quality family planning counselling services.

**Conclusion:**

About 37% of the young women in Sierra Leone do not receive good quality family planning counselling services of which 17.1% received none. Based on the study’s findings, it is important to emphasize the need to ensure that all young women have access to proper counselling services especially for those receiving these services from private health units, from the southern region and richest wealth quintile. Ensuring easier access through increasing affordable and friendly access points and strengthening field health workers’ capacity in providing family planning services could also help improve access to good quality family planning services.

**Supplementary Information:**

The online version contains supplementary material available at 10.1186/s12905-023-02419-8.

## Background

Modern contraceptives are safe and effective methods that can decrease fertility to prevent unplanned pregnancies, in order to help couples, realize their desired family size, and birth to birth intervals [1, 2]. Use of modern contraception is also associated with several non-contraceptive benefits such as contribution to poverty reduction especially at the family level, promotion of gender equity, and prevention of sexually transmitted diseases including the Human Immunodeficiency Virus (HIV) [1]. In addition, the practice of family planning is anchored on the use of modern contraceptives to prevent unplanned pregnancies that are unwanted many of these end up as unsafe abortions in many low resource settings [3]. Therefore, use of modern contraceptives contributes to the progress towards achieving the Sustainable Development Goals (SDGs) by lowering maternal and child morbidity and mortality [2, 4].

Despite global advances in access to family planning services, 214 million women of reproductive age in developing regions of the world still experience an unmet need for modern contraception [3]. In order to achieve the maximum potential of the benefits of the use of family planning methods, consistency in the quality of the service delivery must be prioritized [5, 6]. The quality of family planning services is defined as *“the degree to which, under given technical standards and social values, the available technical and interpersonal means are utilized to achieve specific results of: satisfied users, ample knowledge of the method used, satisfactory levels of acceptance and effective continuous use among the population desiring to space or limit births, adequate method management among users informed of side effects and danger signals”* [7]. The quality of contraceptive counseling that women receive from the health care workers can greatly influence their future contraceptive continuation care-seeking behavior and health outcomes [5, 6]. Quality family planning counseling services have been associated with increased client satisfaction, and a positively influence on the clients’ willingness to adopt and continue using various methods of family planning [8, 9]. Adequate counselling further empowers the women to make an informed, which is a fundamental right [10, 11]. According to the comprehensive reproductive health approach, quality family planning services must be client oriented, empowering women to make informed choices in health care environments that ensure dignity and respect [12, 13].

The fertility rate in Sub Saharan Africa remains high, estimated at 5.1 and Sierra Leone is one of the countries with the worst maternal and child health indicators in the world [14–16]. Furthermore, it is also among the ten countries with high rates of teenage pregnancy in the world [17]. According to the 2019 Sierra Leone Demographic Health Survey (SLDHS), 21% of women aged 15–19 years have begun childbearing; 18% have had a live birth, and 4% are pregnant with their first child with an adolescent fertility rate standing at 102 per 1000 [18]. Of the reported 857 maternal deaths per 100,000 live births in Sierra Leone [17], an estimated 40% occur among teenagers [19]. Studies have partly attributed these poor trends to the country’s post-conflict and Ebola context, in which young women face profound structural exclusion, discrimination and poverty, as well as traditional norms related to gender [20, 21]. Furthermore, Sierra Leone’s health system faces frequent stock outs of medical supplies, limited integration of health services, shortage of skilled health workers and poor remuneration of health workers [22–26]. These challenges negatively affect quality of services. In the post Ebola period, initiatives such as Agenda for Prosperity were promoted to ensure equitable access to quality family planning services which were augmented by earlier initiatives such as the government’s Free Health Care Initiative (FHCI) for children under 5 years of age, pregnant and lactating women [27]. In order to improve quality of health care services, there was high-level political commitment as evidenced by the supply side–strengthening activities such as improved availability of FP commodities and health workers’ capacity building activities and allocating a portion of the Agenda for Prosperity development plan to family planning services [27].

Although a number of strategies have been adopted to address the various challenges, including the scaling up of family planning services, uptake is still low [18, 27]. The contraceptive prevalence rate (CPR) among all women and currently married women is 24% and 21% respectively with more than one out of every three women (35%) who begin using a contraceptive method discontinue the method within 12 months [18]. Side effects/health concerns remains the commonest reason for discontinuation, accounting for 16% of all the cases yet these can be fully discussed during family planning counselling sessions [18]. These health concerns are commonly due to inadequate family planning counselling content which is worsened by limited alternative sources of information as evidenced by limited exposure of mass media family planning messages in Sierra Leone [18]. Over 69% of all women and 73.3% of adolescents have no exposure to family planning messages across all the four media sources (television, radio, mobile phones and newspapers) [18]. Furthermore, young women in Sierra Leone have the lowest knowledge of the fertile period (27.8% compared to the national average of 37%) and have the highest unmet need for family planning yet over 40% of the country’s maternal deaths occur among teenagers [18]. Given that a third of women discontinue family planning methods within twelve months of which the young women contribute a great proportion with side effects/health concerns contributing the greatest proportion [18], efforts to strengthen, expand access, decrease discontinuation and increase up take of family planning services among young women must focus on improving the quality of family planning counselling services. Previous research on family planning in Sierra Leone, has focused on the effect of Ebola and the civil conflict on health services, the government’s efforts during the post Ebola and conflict periods, contraception uptake among women of reproductive age with a dearth of information on young women especially on the topic of quality of family planning counselling services. Therefore, this study determined the quality of family planning counselling information and its determinants among young women in Sierra Leone, using the 2019 SLDHS data.

## Methods and materials

### Data source and sampling procedure

This was a cross-sectional study based on secondary data from the 2019 SLDHS, which was conducted from May 2019 to August 2019. This was a nationally representative survey of a weighted sample of 15,574 women aged 15–49 years. Our analysis included all young women aged 15 to 24 years who were currently using a contraceptive. All women aged 15–24 that were either permanent residents of the households in the sample or visitors present in the household on the night before the survey were eligible to be interviewed. Out of the 15,574 women, 6,055 women were aged 15 to 24 years and 1,506 of these were currently using a family planning method. A full protocol with detailed explanation about the data collection process and sampling is available online [18].

### Variables

#### Outcome variables

Quality of family planning counselling services was the outcome and this was a composite outcome assessed basing on the responses to the questions that form the basis of the Method Information Index (MII) [28, 29]. Good quality was defined as family planning counselling services that included a woman being told about side effects, how to deal with side effects and about other family planning methods. Each of the three indicators of quality scored one and the total score was 3 [8, 28, 30]. Score of 3 indicated good quality and was coded as one (1) while score of 2 and below indicated poor quality and was coded as zero (0).

### Covariates

Based on the literature and available data, we adopted Andersen’s behavioral model of health service use [1, 8, 31] as shown in Table 1. In this model, utilization of healthcare is a function of three major elements: predisposing factors, enabling factors and healthcare needs [32]. The predisposing factors in the model were: age, level of education, region of residence, place of residence, religion, sex of household head and marital status. Wealth index, working status, exposure to family planning mass media messages, being visited by a field health worker, problems seeking permission, access to internet, source of family planning method and distance to the nearest health facility as an indicator of access were considered as enabling factors, while having visited a health facility within the last 12 months was included in the model as a proxy for the need factor, as illustrated in Additional file 1 [32, 33].

**Table 1:** Categorization of independent variables

Variable	Categorization	
Exposure to family planning mass media messages	Yes and No	Composite variable where exposure to family planning messages either on radio or television or newspapers or phone texts was a yes
Source of family planning method	Government and private health facilities	-
Access to internet	Yes and No	-
Age	15 to 19 years and 20 to 24 years	-
Residence	Urban and Rural	-
Region	Northern, Eastern, Southern, Western and Northwestern	-
Religion	Muslims and Christians and others	-
sex of household head	Male and female	-
level of education	No education, primary, secondary and tertiary	-
Working status	Yes and No	-
Wealth index	Richest, richer, middle, poorer and poorest quintiles	-
Having visited a health facility within the last 12 months	Yes and No	-
Having been visited by a field health worker within the last 12 months	Yes and No	-
Problems seeking permission and distance to health facility	No big problems and Big problems	SLDHS had three original categories (no problem, no big problem and big problem) however, after data collection, no woman reported no problem
Problems with distance to nearest health facility	No big problems and Big problems	SLDHS had three original categories (no problem, no big problem and big problem) however, after data collection, no woman reported no problem
Marital status	Married and Not married	Married included both formal and informal unions

### Statistical analysis

In order to account for the unequal probability sampling in different strata [34] and to ensure representativeness of the study results [35], DHS sample weights were applied. SPSS version 25.0 statistical software complex samples package incorporating the following variables in the analysis plan to account for the multistage sample design inherent in the DHS dataset: individual sample weight, sample strata for sampling errors/design, and cluster number was used [36–38]. Tabulation for independent variables was done for proportions and frequencies. Bivariable logistic regression was done to assess the association of each independent variable with quality of family planning services and crude odds ratio (COR), 95% confidence interval (CI) and p-values are presented. Independent variables found significant at bivariable level with p-values less than 0.25 were added in the multivariable logistic regression model. Adjusted odds ratios (AOR), 95% Confidence Intervals (CI) and p-values were calculated with statistical significance level set at p-value < 0.05 [1]. All variables in the model were assessed for collinearity, which was considered present if the variables had a variance inflation factor (VIF) greater than 3.

## Results

A total of 1,506 young women were included in the study (Table 2). Of these, 955 (63.4%, 95% CI: 60.5–65.3) received good quality family planning counselling services (Additional file 2). Majority of the young women had no exposure to family planning mass media messages (61.6%), received family planning services from government health facilities (78.1%), were not married (79.0%), had secondary level of education (76.7%), were aged 20 to 24 years (52.2%) and resided in urban areas (56.3%).

**Table 2:** Socio-demographic characteristics of young women in Sierra Leone as per the 2019 SLDHS

Characteristics	N = 1,506	%
**Heard family planning messages on mass media**		
No	928	61.6
Yes	578	38.4
**Source of family planning method**		
Government health facility	1176	78.1
Private health facility	330	21.9
**Access to internet**		
No	1129	74.9
Yes	377	25.1
**Age**		
15 to 19	720	47.8
20 to 24	786	52.2
**Residence**		
Urban	847	56.3
Rural	658	43.7
**Region**		
Western	338	22.5
Eastern	288	23.9
Northwestern	243	16.1
Northern	360	23.9
Southern	276	18.3
**Religion**		
Islam	1084	72.0
Christianity and others	421	28.0
**Sex household head**		
Male	946	62.8
Female	560	37.2
**Working status**		
Not working	752	49.9
Working	754	50.1
**Education Level**		
No Education	147	9.7
Primary Education	147	9.7
Secondary Education	1155	76.7
Tertiary	58	3.8
**Wealth Index**		
Poorest	160	10.6
Poorer	214	14.2
Middle	315	20.9
Richer	456	30.3
Richest	362	24.0
**Visited health facility within 12 months**		
No	718	47.7
Yes	788	52.3
**Visited by field health worker within 12 months**		
No	1062	70.5
Yes	444	29.5
**Permission to access healthcare**		
Big problem	316	21.0
Not big problem	1190	79.0
**Distance to health facility**		
Big problem	547	36.3
Not big problem	959	63.7
**Marital status**		
Not married	1189	79.0
Married	317	21.0

### Factors associated with quality of family planning counselling services

After adjusting for other variables, factors that were statistically associated with good quality of family planning counselling services were receiving family planning services from government health facilities (aOR: 2.50, 95% CI: 1.83–3.41), residing in the Southern region (AOR: 0.39, 95% CI: 0.22–0.69), belonging to the richest wealth quintile (aOR: 0.49, 95% CI: 0.24–0.98), having visited a health facility within the last 12 months (aOR: 1.93, 95% CI: 1.45–2.58), having been visited by a health field worker within the last 12 months (aOR: 1.67, 95% CI: 1.24–2.26) and having no major challenges with distance to access healthcare (aOR: 1.45, 95% CI: 1.10–1.90). The details are shown in Table 3.

**Table 3:** Factors associated with quality of family planning services among young women in Sierra Leone

Characteristics	Crude model cOR (95% CI)	P-value	Adjusted model aOR (95% CI)	P-value
**Heard family planning messages on mass media**				
No	1		1	
Yes	1.22 (0.92–1.64)	0.172	1.21 (0.89–1.63)	0.229
**Source of family planning method**				
Private health facility	1			
Government health facility	**3.09 (2.33–4.11)**	**< 0.001**	**2.50 (1.83–3.41)**	**< 0.001**
**Access to internet**				
No	1		1	
Yes	0.71 (0.51–1.01)	0.054	0.95 (0.65–1.40)	0.792
**Age**				
15 to 19	1		1	
20 to 24	0.85 (0.66–1.10)	0.215	0.97 (0.74–1.28)	0.84
**Residence**				
Rural	1		1	
Urban	**0.60 (0.44–0.81)**	**0.001**	0.86 (0.56–1.32)	0.491
**Region**				
Western	1		1	
Southern	0.65 (0.40–1.05)	0.079	**0.39 (0.22–0.69)**	**0.001**
Northwestern	1.43 (0.83–2.47)	0.194	0.73 (0.40–1.31)	0.291
Northern	**2.08 (1.26–3.41)**	**0.004**	1.05 (0.59–1.87)	0.86
Eastern	1.28 (0.76–2.16)	0.360	0.66 (0.37–1.19)	0.164
**Religion**				
Islam	1		1	
Christianity and others	**0.76 (0.58–0.99)**	**0.042**	0.89 (0.65–1.21)	0.459
**Sex household head**				
Male	1		-	
Female	0.90 (0.69–1.18)	0.438		
**Working status**				
Not working	1		1	
Working	**1.46 (1.11–1.91)**	**0.007**	1.07 (0.77–1.48)	0.689
**Education Level**				
No Education	1		1	
Primary Education	**0.56 (0.32–0.98)**	**0.043**	0.58 (0.32–1.04)	0.069
Secondary Education	0.68 (0.44–1.05)	0.080	0.87 (0.54–1.40)	0.570
Tertiary	**0.33 (0.14–0.76)**	**0.010**	0.77 (0.31–1.93)	0.578
**Wealth Index**				
Poorest	1		1	
Poorer	1.05 (0.61–1.81)	0.866	1.11 (0.65–1.89)	0.703
Middle	1.42 (0.84–2.40)	0.194	1.36 (0.81–2.30)	0.244
Richer	0.81(0.48–1.37)	0.431	0.99 (0.55–1.79)	0.968
Richest	**0.49 (0.28–0.86)**	**0.012**	**0.49 (0.24–0.98)**	**0.043**
**Visited health facility within 12 months**				
No	1		1	
Yes	**2.28 (1.74–2.98)**	**< 0.001**	**1.93 (1.45–2.58)**	**< 0.001**
**Visited by field health worker within 12 months**				
No	1		1	
Yes	**2.29 (1.71–3.06)**	**< 0.001**	**1.67 (1.24–2.26)**	**0.001**
**Permission to access healthcare**				
Big problem	1		-	
Not big problem	1.06 (0.73–1.52)	0.775		
**Distance to health facility**				
Big problem	1		**1**	
Not big problem	1.23 (0.92–1.64)	0.154	**1.45 (1.10–1.90)**	**0.008**
**Marital status**				
Not married	1		-	
Married	1.02 (0.74–1.39)	0.907		

## Discussion

This analysis reveals that about 36.6% of young women received inadequate family planning counselling including 17.1% that received no counselling. About two thirds of the young women received good quality family planning counselling services. The determinants identified were; receiving family planning services from government health facilities, residing in the southern region, belonging to the richest wealth quintile, having visited a health facility within the last 12 months, having been visited by a health field worker within the last 12 months, and having no major challenges with distance to access healthcare.

In Sierra Leone, proportion of younger women receiving good quality FP counselling services is higher compared to findings from countries in Sub-Saharan Africa such as Kenya (56.7%), Uganda (34.9%) and Senegal (18%) [8, 29, 39]. Furthermore, studies from other regions in countries such as Nepal and Spain indicated that the quality of FP counselling services were low and unsatisfactory based on the patient expectations and experience thus demanding for more information about the different methods [40, 41]. The difference in the findings could probably be due to different study designs and populations. The current study focused on young women, who had participated in a nationally representative cross-sectional survey, while the other studies included women of all age groups and they did not use a nationally representative sample. We could not find a similar study for direct comparison.

Young women who received family planning counselling from government facilities had higher odds of receiving all the contents of counselling compared to their counterparts in private facilities. Previous studies have shown contradicting results, in Kenya no association was reported between the type of facility and quality of FP counselling [29]. On the other hand, studies from Jordan have reported lower odds of adequate counselling among government facilities [10]. However, some studies from Uganda, Ethiopia and Kenya, report findings similar to ours [28, 39, 42]. The predominant source of modern contraceptive methods in Sierra Leone is the public sector providing over 80% of all clients while the private medical sector covers only 18% of users [18]. Among the 18% clients who seek care from the private sector, the majority (12%) get it from pharmacies/drug shops [18]. Since pharmacies are usually staffed with healthcare workers who are usually not well trained in family planning counselling [43], this might partly contribute to the lower odds of adequate counselling observed among young women seeking care from private facilities. Although studies have shown that women might prefer private providers for confidentiality and respectful treatment, women have further noted that private providers prioritize profit over safe medical practice [42–44]. Hence, these end up hiring less experienced family planning providers who are less likely to offer comprehensive counseling and also stock sub-standard products [42, 43].This might also partly explain why women in the richest wealth quintile had lower odds of receiving quality FP counseling services, since they are more likely to utilize private family planning providers, where the waiting time to access a service is shorter [42]. Therefore, there is need for stronger public private partnerships under which the Sierra Leone ministry of health can build capacity of family planning services providers in private health facilities to ensure that women get god quality family planning services.

Women from the southern region had less odds of receiving adequate family planning counselling compared to those from the western region. Region has been shown in Ethiopia to have an association with quality of family planning services [28]. The western region is the most economically developed with a high concentration of skilled healthcare providers and health facilities that are easily accessible hence easier access to good quality family planning counselling services [45, 46]. Furthermore, the documented closer relationships women in the southern region have with traditional health care providers such as traditional birth attendants might also reflect poor health seeking behaviors hence being more likely to access care from informal settings leading to receiving services of poor quality [15]. There is need to further sensitize these women on the advantages of accessing care through qualified providers in health facilities and in the community health programme with community-based field health workers.

Access to health facilities has been shown to be a significant factor in the quality of family planning counseling as demonstrated by the high odds of adequate counseling among women who had no big problems with distance to nearby health facility, those had visited a health facility and been visited by a field health worker within 12 months. All these factors increase the contact hours between the young women and health workers’, which allows enough to give information and answer questions related to FP [39]. It also improves the provider patient relationship, leading to trust and continued use of the selected method of contraception.

### Strengths and limitations

The novelty of this study stems from the fact that, it is the first of its kind to have investigated quality of family planning counselling among young women using the latest nationally representative data. However, it is not without some limitations, the data were collected based on women’s self-reports. This is usually prone to recall and social desirability bias. Second, the cross-sectional nature of the study does not permit causal inferences to be made. The cadre/qualification of the provider’s and the clients’ experiences and satisfaction at the health facility level as key indicators of quality counselling services were not assessed. The use of self-reported answers and the possibility of giving false answers due to social desirability risks recall and information bias.

## Conclusion and public health implications

More than two thirds (63.4%) of young women received good quality family planning counselling services. Given the low modern contraceptive utilization rate in Sierra Leone that is lower than the discontinuation prevalence, maternal health stakeholders need to prioritize programmes aimed at ensuring quality of family planning services. From this study, health system related factors have shown to have the greatest association with quality of care. There is need to bring services closer to women through empowering field health workers to provide family planning to mitigate the effect of distance as a barrier to access of health services. Since having visited a health facility showed higher odds of receiving good quality family planning services, there is need to integrate family planning counselling in all major service points in health facilities to reduce on missed opportunities. Further research should focus on understanding the barriers and challenges in the provisioning of good counseling to young women mainly in private health facilities, richest households and in the southern region of Sierra Leone.

## Electronic supplementary material

Below is the link to the electronic supplementary material.

**Supplementary File 1**: Independent and outcome variables mapped on to the theoretical framework: Andersen’s Behavioral Model of Health Service Use.

**Supplementary File 2**: Frequency of components of family planning counselling services among young women in Sierra Leone.

## Data Availability

The data set used is openly available upon permission from MEASURE DHS website (URL: https://www.dhsprogram.com/data/available-datasets.cfm).

## Abbreviations

CI: Confidence Interval
COR: Crude Odds Ratio
DHS: Demographic Health Survey
SLDHS: Sierra Leone Demographic Health Survey
OR: Odds Ratio
SPSS: Statistical Package for Social Science
