# Supplementary material for: Determinants of quality contraceptive counselling information among young women in Sierra Leone: insights from the 2019 Sierra Leone demographic health survey
Source: BMC Womens Health. 2023 May 15;23:266. doi: 10.1186/s12905-023-02419-8 (PMC10186652; doi:10.1186/s12905-023-02419-8)
Supplement: Supplementary file 1 — Independent and outcome variables mapped on to the theoretical framework: Andersen’s Behavioral Model of Health Service Use. [file 12905_2023_2419_MOESM1_ESM.docx]

**Environmental factors**

-Health care systems

-Access to healthcare (distance and seeking permission)

**Outcome**

Quality of family planning counselling information

**Population characteristics**

**-Predisposing factors**: Demographics (age, education, marital status, region, sex of household head, residence)

-**Enabling factors**: working status, being visited by a field health worker, wealth index, exposure to mass media, access to internet, source of family planning method, seeking permission and distance to seek healthcare

-**Need**: Having visited a health facility within the last 12 months

**Supplementary File 1:** Independent and outcome variables mapped on to the theoretical framework: Andersen’s Behavioral Model of Health Service Use.
