# Supplementary material for: Determinants of quality contraceptive counselling information among young women in Sierra Leone: insights from the 2019 Sierra Leone demographic health survey
Source: BMC Womens Health. 2023 May 15;23:266. doi: 10.1186/s12905-023-02419-8 (PMC10186652; doi:10.1186/s12905-023-02419-8)
Supplement: Supplementary file 2 — Frequency of components of family planning counselling services among young women in Sierra Leone. [file 12905_2023_2419_MOESM2_ESM.docx]

**Frequency of components of family planning counselling services among young women in Sierra Leone**

|  | **Frequency**  **N=1506** | **%** | **95% CI** |
| --- | --- | --- | --- |
| **No counselling** | 256 | 17.1 | 15.2-19.0 |
| **One counselling component** | 184 | 12.2 | 11.1-14.4 |
| **Two counselling components** | 111 | 7.3 | 6.1-8.7 |
| **All the three counselling components** | 955 | 63.4 | 60.5-65.3 |

CI= Confidence Interval, %= Percentage
